# Stereotactic body radiotherapy as a viable treatment on extracranial oligometastases in melanoma patients: a retrospective multicentric study

**DOI:** 10.3389/fonc.2024.1322515

**Published:** 2024-03-05

**Authors:** Victorine Trentesaux, Sophie Maiezza, Emilie Bogart, Marie-Cécile Le Deley, Emmanuel Meyer, Ludovic Vanquin, David Pasquier, Laurent Mortier, Xavier Mirabel

**Affiliations:** ^1^ Academic Department of Radiation Oncology, Centre Oscar Lambret, Lille, France; ^2^ Department of Dermatology, Hôpital Claude Huriez du Centre hospitalo-universitaire (CHU) de Lille, Lille, France; ^3^ Clinical Research and Innovation Department, Centre Oscar Lambret, Lille, France; ^4^ Department of Radiotherapy, Centre Francois Baclesse, Caen, France; ^5^ Department of Medical Physics, Centre Oscar Lambret, Lille, France; ^6^ Centre de Recherche en Informatique, Signal et Automatique de Lille (CRIStAL), Centre national de recherche scientifique (CNRS-UMR) 9189, University of Lille, Lille, France; ^7^ Department of Medicine, University of Lille, Lille, France

**Keywords:** extracranial oligometastases, melanoma, stereotactic body radiotherapy, SBRT, CyberKnife

## Abstract

**Introduction:**

Stereotactic radiotherapy (SBRT) potentially has a role in the management of oligometastatic melanoma. However, literature with data specific to this management is very limited. The objectives of this study were to evaluate the time to local control (LC) of extra-cranial melanoma metastases after SBRT treatment and to help establish if SBRT is a useful therapy for oligometastatic melanoma.

**Methods:**

A retrospective study was conducted with data collected from two referral centers in France between 2007 and 2020. The oligometastatic status of patients was reported based on the latest recommendations with a maximum of three lesions prior to treatment.

**Results:**

A total of 69 patients receiving SBRT for 88 oligometastatic melanoma metastases were included. The median follow-up time was 42.6 months. Most patients were treated for metachronous oligometastatic lesions. Occurrence of oligoprogression, oligorecurrence, and oligopersistence was reported in 42.0%, 39.1%, and 17.4% of cases, respectively. Treated lesions were mostly pulmonary (40.6%), followed by lymph node (34.8%) and hepatic sites (24.6%). Progression-free survival at 1, 2, and 3 years were 47.0% (35-59), 27.0% (16-39), and 25.0% (15.0-37.0), respectively. Time to LC rates at 1, 2, and 3 years were 94.2% (87.0-98.1), 90.3% (81.3-96.1), and 90.3% (81.3-96.1), respectively. Overall survival at 1, 2, and 3 years were 87% (76.0-93.0), 74.0% (76.0-93.0), and 61.0% (47.0-73.0), respectively. Only 17.4% of patients experienced acute, grade 1 or grade 2 toxicities with no reports of grade 3 or higher toxicities.

**Conclusion:**

SBRT demonstrated efficacy in managing melanoma patients with extracranial oligometastases and showed an overall low toxicity profile. Future randomized studies are needed to establish the role of SBRT in therapeutic approaches for patients with oligometastatic melanoma.

## Introduction

Melanoma accounts for approximately 10% of all skin cancers and approximately 2 to 3% of all cancers. It is responsible for nearly 90% of skin cancer related deaths (1). Historically, metastatic melanoma was associated with a poor prognosis and median survival periods of only 8 to 12 months (2, 3). With the emergence of targeted therapies and immunotherapies, there has been a significant shift in the management of metastatic melanoma which median OS (minimum follow-up 6,5 years) was 72.1 months with the combination of IPILIMUMAB and NIVOLUMAB (4).

The concept of the oligometastatic state was initially described in 1995 as an intermediate stage between locoregional involvement and systemic disease that curatively could be treated if the primary and secondary lesions are addressed. During this stage, metastases are limited in number and usually involve less than five lesions (5, 6). However, despite the clinical significance of oligometastatic diseases (OMDs), lack of clinically available biomarkers makes diagnosis challenging. Because of this, OMD diagnosis is primarily based on imaging, potentially leading to different therapeutic scenarios based on the evolution of the pathology and prognosis. Moreover, ESTRO-EORTC have created nomenclature for OMDs in an effort to help clinicians identify the type of OMD a patient presents with (7).

Local ablative therapy (LAT) for oligometastatic patients presents an opportunity to improve local control (LC), defer the initiation of systemic therapy, elicit a complete response, and extend overall survival (OS). Stereotactic body radiotherapy (SBRT), which delivers high biologically equivalent radiation doses with pinpoint accuracy to small tumor volumes, has been incorporated as a component of focal therapy in tandem with surgery and radiofrequency radiation. Randomized trials focusing on lung, prostate and breast cancers have provided evidence that LAT using SBRT significantly enhances LC rates and promotes prolonged survival thus supporting the clinical relevance of this treatment strategy for patients with oligometastatic cancer (8–14).

SBRT could limit or delay the need for systemic therapies in case of oligorecurrence, maintain current lines of treatment by eliminate escape lesions that have lost sensitivity to the systemic therapy in case of oligoprogression, or serve as a closing treatment to stop systemic therapy in case of oligopersistence.

To date, most literature on the application of LAT with SBRT in melanoma have focused on the management of brain metastases. Moreover, available data on the treatment of extracranial metastases is very limited (15). Retrospective studies by Franceschini et al. (2017) with 31 patients, and Kropp et al., 2016 with 16 patients, are among the few that focus on the field (16, 17). In other studies, Stinauer et al. (2011) reported data on 17 patients who had oligometastatic melanoma (18) and Guckenberger et al. (2020) included data on 15 cases of melanoma metastases even though 525 secondary pulmonary lesions were investigated (7). The objective of this study was to evaluate the contribution of SBRT to the management of extracranial oligometastatic melanoma.

## Materials and methods

A multicentric retrospective cohort study was conducted at two cancer centers in France (the Oscar Lambret Center in Lille and the François Baclesse Center in Caen) between 2007 and 2020. Inclusion criteria were patients who had: i) histologically confirmed oligometastatic melanoma, ii) aged 18 or older, iii) receiving treatment with SBRT administered to at least one secondary lesion in the pulmonary, hepatic or lymph node sites, and iv) a maximum of three simultaneous metastases to consider the oligometastatic character. Patients with treated and controlled intracranial disease were included. Diagnosis of secondary lesions was based on imaging tests such as CT, MRI or PET scans. Patients with an Eastern Cooperative Oncology Group (ECOG) Performance Status Scale <2 and a life expectancy of at least three months were also included. Exclusion criteria included patients with more than three simultaneous metastases as well as those who received non-stereotactic irradiation of their metastases.

SBRT was administered to treat metastatic lesions using the Cyberknife robotic linear accelerator. A 4D scanner for ITV was used for more recently treated pulmonary lesions. Synchrony software was used to track moving targets such as secondary hepatic lesions with the help of fiducials implanted in or near the target lesion. The prescription isodose was 80%. Systemic or local treatment of metastases were administered before or during SBRT. The oligometastatic status of the patients was subsequently classified based on ESTRO-EORTC recommendations (7).

We differentiated between induced OMD (patient have history of polymetastatic disease and OMD is induced by systemic treatment) and genuine OMD (no history of polymetastatic disease before OMD). For each category we define then oligorecurrence, oligoprogression and oligopersistence.

Oligorecurrence was occurrence of OMDs during an interval without systemic treatment. Oligoprogression was the development of the OMD under systemic treatment. Oligopersistence was stable or partially responding disease on imaging while on (or after) systemic therapy (**Supplementary Figure 1**).

The primary endpoint was time to LC, which was defined as the time to progression between the start of SBRT and the first local progression. Systemic progression and death were considered competitive events. Secondary endpoints, determined retrospectively, included progression-free survival (PFS), OS, toxicity. Progression-free survival (PFS) was estimated per patient from the date of SBRT initiation to the date of progression or death from any cause. The date of progression was estimated from radiological reports demonstrating tumour progression. A sensitivity analysis was performed by estimating PFS excluding patients with targeted/chemotherapy. Dosimetry data were collected according to the criteria indicated in Report 91 published by the International Committee on Radiologic Units (ICRU) (19). Statistical analysis was performed using Stata^®^ software, Version 15.0 (StataCorp LLC College Station, TX, USA) and included univariate Cox models to test the prognostic value of factors on OS, PFS, and time to LC. The proportional hazards assumption of the Cox model was tested for each variable by the Schoenfeld residuals test.

## Results

**Figure 1:**
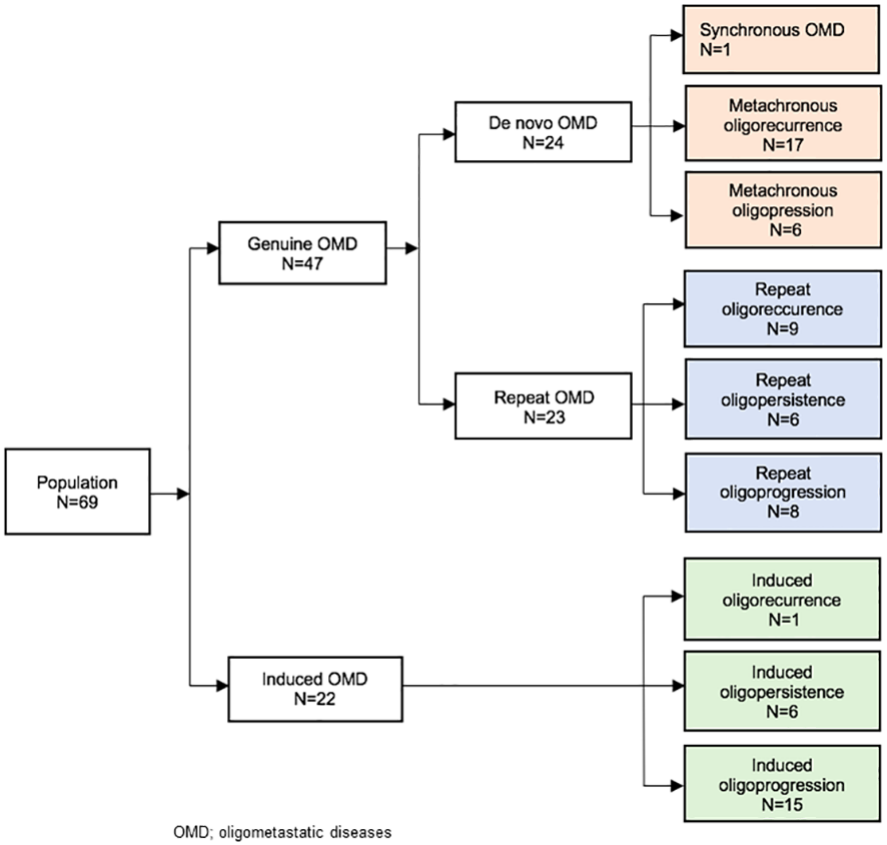
Study flow chart according to the model of Guckenberger et al. (2020) (7).

In total, 69 patients with oligometastatic melanoma were included in the study. According to TNM classification, 8^th^ edition, most patients had T3 (24.6%) and T4 (30.4%) primary tumors, and most were N0 (62.3%) and M0 (87.0%) (**Table 1**). Nine patients had synchronous metastatic disease. SBRT was used to treat 88 lesions, with most patients receiving treatment for one (76.8%) or two (18.8%) metastases. The treated lesions were located primarily in the pulmonary (40.6%), lymph node (34.8%) and hepatic (24.6%) sites. Regarding treatment indications, 42% of patients were treated for oligoprogression, 39.1% for oligorecurrence and 17.4% for oligopersistence (**Figure 1**; **Table 2**). Systemic therapy was used in combination with SBRT in 68% of cases. Immunotherapy was the most commonly employed systemic therapy (76.6%). The prescribed median total dose was 45Gy (Min-Max: 15-60), with most patients receiving 3 fractions and a median dose per fraction of 15Gy (Min-Max: 5-20). The standard regimen used for hepatic lesions was 45Gy in three fractions of 15Gy, for pulmonary lesions, 54Gy in 3 fractions of 18Gy and for lymph node lesions, 45Gy in three fractions of 15Gy. Considering the 88 lesions treated, the median D98%, D50% and D2% of the PTV were respectively 43.0 (Min-Max: 13.0-66.5) Gy, 49.2 (16.4;74.4) Gy and 53.5 (Min-Max: 17.8-81.6) Gy (**Table 2**, **Supplementary Table 1**). Except for one patient, all were treated for metachronous oligometastatic lesions. In total, 68.1% of patients had genuine OMDs and 31.9% had induced OMDs (**Figure 1**). According to the ESTRO-EORTC recommendations, the two most common oligometastatic statuses were genuine OMDs (24.6%) in oligorecurrence and induced OMDs (15 patients, 21.7%) in oligoprogression.

**Table 1:** Demographic and baseline characteristics of 69 melanoma patients at two cancer centers in France (the Oscar Lambret Center in Lille and the François Baclesse Center in Caen) between 2007 and 2020.

		Total n (%)	Mean ± SD	Median (Min-Max)	Missing Data
**Gender (male)**		38 (55.1)			
**Age at diagnosis**		38	58.5 ± 13.9	58 (19-81)	
Pathology					
*Histological type*		59			10
	*Superficial Spreading Melanoma (SSM)*	19 (32.2)			
	*Nodular*	15 (25.4)			
	*Acrolentiginous*	3 (5.1)			
	*Dubreuilh*	2 (3.4)			
	*Mucosal*	5 (8.5)			
	*Choroidal*	2 (3.4)			
	*Desmoplastic*	2 (3.4)			
	*Achromic*	1 (1.7)			
	*Unclassifiable*	10 (16.9)			
*Breslow (mm)*		56	4.3 ± 3.8	2.9 (0.4-18.0)	13
**Tumor staging**		69			0
*Stage I*		6 (8.7)			
*Stage II*		12 (17.4)			
*Stage III*		17 (24.6)			
*Stage IV*		21 (30.4)			
*Stage x*		13 (18.9)			
**Lymph node staging**		69			0
*Stage 0*		43 (62.3)			
*Stage I*		14 (20.3)			
*Stage II*		3 (4.3)			
*Stage III*		8 (11.6)			
*Stage x*		1 (1.4)			
**Metastasis staging**		69			0
*Stage 0*		60 (87.0)			
*Stage Ia*		3 (4.3)			
*Stage Ib*		4 (5.8)			
*Stage Ic*		2 (2.9)			
**BRAF status of the primary melanoma lesion**		69			0
*Unmutated*		35 (50.7)			
*Mutated*		11 (15.9)			
*Unknown*		23 (33.3)			
Initial treatment					
*Initial surgery*		66 (95.7)			
	*Time from diagnosis*		0.6 ± 1.2	0.0 (0.0-5.6)	
*Initial radiotherapy*		16 (23.2)			
	*Time from diagnosis*		3.7 ± 2.6	3.6 (0.0-10.0)	
*Systemic therapy*		14 (20.3)			
	*Time from diagnosis*		6.2 ± 12.5	2.4 (0.0-49.0)	
	*Duration of treatment (months)*		17.9 ± 17.1	12.8 (2.3-62.6)	

SD, standard deviation.

**Table 2:** Characteristics of oligometastatic disease treatment and dosimetric data.

		Total n (%)	Mean ± SD	Median (Min-Max)	Missing Data
ECOG at the beginning of treatment					
*0*		49 (71.0)			
*1*		18 (26.1)			
*2*		2 (2.9)			
**Time from initial diagnosis to start SBRT (years)**			5.1 ± 4.2	3.8 (0.4-18.4)	
Number of treated lesions per patient					
*1*		53 (76.8)			
*2*		13 (18.8)			
*3*		3 (4.4)			
Total number of metastases per patient at time of SBRT					
*1*		37 (53.6)			
*2*		22 (31.9)			
*3*		10 (14.5)			
OMD indication for Cyberknife					
*Oligoprogression*		29 (42.0)			
*Oligopersistence*		12 (17.4)			
*Oligorecurrence*		27 (39.1)			
*Synchronous oligometastatic disease*		1 (1.4)			
**Systemic therapy combined with SBRT**		47 (68.2)			
*Type of systemic therapy*		47			
	*Chemotherapy*	6 (12.8)			
	*Targeted therapy*	5 (10.6)			
	*Immunotherapy*	34 (72.3)			
	*Chemotherapy + Immunotherapy*	2 (4.3)			
Temporary discontinuation of systemic therapy*		3 (6.4)			
*Duration of treatment (month)*		36	16.3 ± 12.9	13.5(1.0-62.6)	10
SBRT characteristics					
*SBRT site*		69			
	*Hepatic*	17 (24.6)			
	*Pulmonary*	28 (40.6)			
	*Lymph node*	24 (34.8)			
*Lesion size (mm)*		59	20.1 ± 14.1	15(4-76)	10
*Total dose received (Gy)*			44.4 ± 11.8	45 (15-60)	
*Dose per fraction (Gy)*			13.5 ± 4.1	15 (5-20)	
*Duration of SBRT (days)*			8.6 ± 3.0	8 (1-18)	
*Dosimetric data (lesions)*		88			
	*PTV D98*		40.1 ± 10.2	43.0(13.1-66.5)	
	*PTV D50*		46.2 ± 11.3	49.2(16.4-74.4)	
	*PTV D2*		50.8 ± 12.6	53.5(17.8-81.6)	

*Temporary cessation was observed for three patients corresponding to a duration of 10, 14 and 30 days. ECOG; Eastern Cooperative Oncology Group, SBRT; Stereotactic Body Radiotherapy, Gy; Gray, PTV; Planned Target Volume. OMD, oligometastatic diseases.

**Figure 2:**
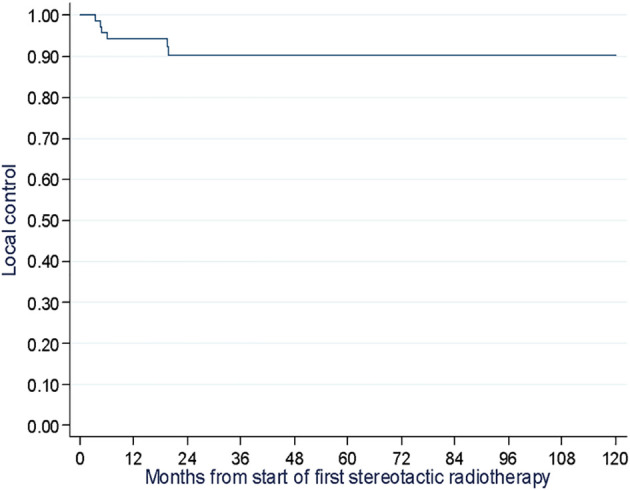
Time to local control assessment of 69 melanoma patients at two cancer centers in France (the Oscar Lambret Center in Lille and the François Baclesse Center in Caen) between 2007 and 2020.

The median follow-up time was 42.6 months (Min-Max: 4.6-126.8). Time to LC at 1, 2 and 3 years was 94.2% (Min-Max: 87.0-98.1), 90.3% (Min-Max: 81.3-96.1) and 90.3% (Min-Max: 81.3-96.1), respectively (**Figure 2**).

**Figure 3:**
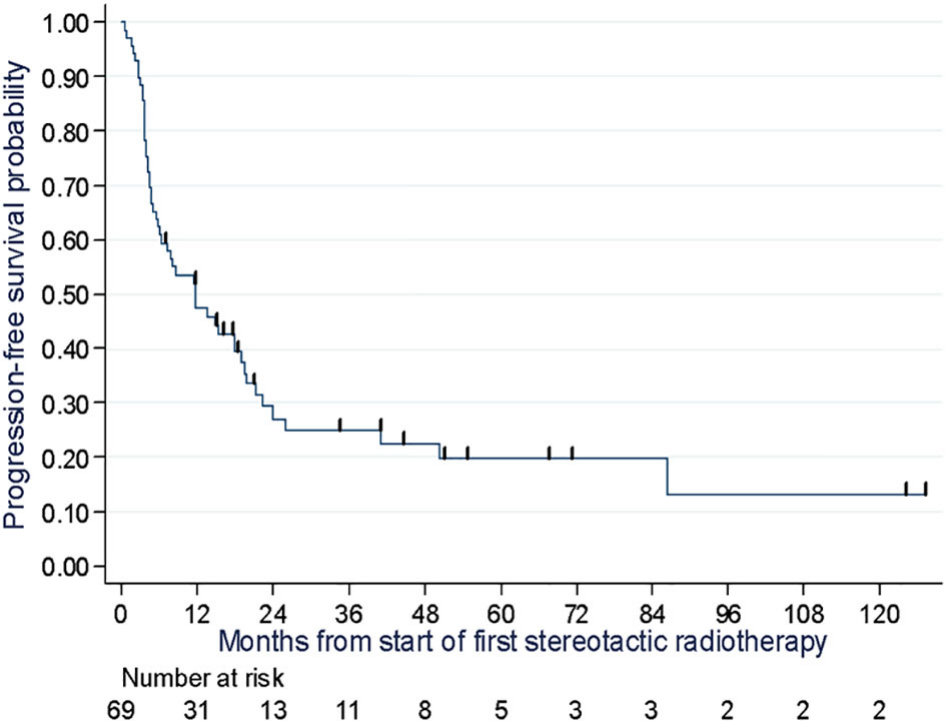
Progression free survival probability assessment of 69 melanoma patients at two cancer centers in Lille and Caen in France between 2007 and 2020.

**Figure 4:**
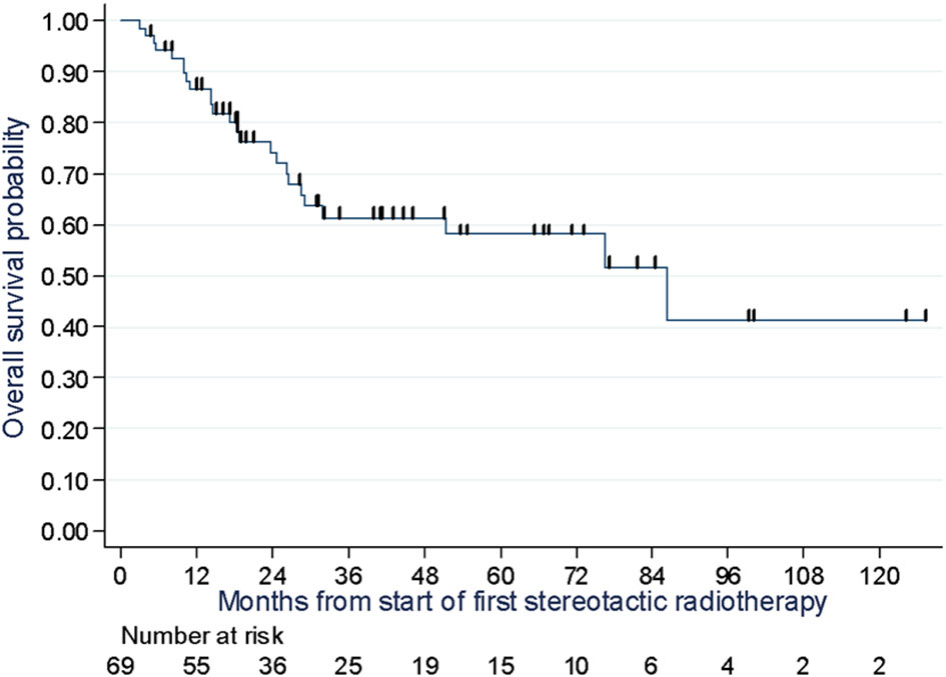
Overall survival assessment of 69 melanoma patients at two cancer centers in Lille and Caen in France between 2007 and 2020.

Out of the total 51 reported progressions, two were local, four were local and systemic, 44 were systemic and one was recorded as death with no prior progression. Most of the systemic progressions occurred in patients treated at lymph node (n=18) and pulmonary (n=17) sites. The median PFS was 11.8 months (95% CI: 6.0 to 19.0), and the PFS rates at one, two, and three years were 47.0% (Min-Max: 35.0-59.0), 27% (Min-Max:16.0-39.0), and 25.0% (Min-Max: 15.0-37.0), respectively (**Figure 3**). PFS was similar when excluding the 11 patients with targeted/chemotherapy: the median PFS was 11.4 months (95% CI: 6.0 to 18.0), and the PFS rates at one, two, and three years were 44.0%, 29%, and 26.0% (sensitivity analysis, **Supplementary Figure 2**). At the time of analysis, 25 deaths due to melanoma were reported, with a median OS of 86.5 months (95% CI: 29.2 months not reached). The 1-year, 2-year, and 3-year OS rates were 87.0% (Min-Max: 76.0-93.0), 74.0% (Min-Max: 76.0-93.0), and 61.0% (Min-Max: 47.0-73.0), respectively (**Figure 4**). Of the 69 patients, 13 were alive and free of progression at 24 months after SBRT. It was oligorecurrence OMDs in 6 patients, oligoprogression OMDs in 4 patients and oligopersistence in 3 patients.

In 68.0% of patients, SBRT was administered alongside systemic therapy. After relapse, the treatment was modified in approximately 62% of patients, with a median delay of 5.7 months. In 32% of patients, SBRT was administered as a stand-alone treatment without systemic therapy. Subsequently, systemic therapy was initiated in 12 patients, with a median delay of 5.2 months after relapse. Immunotherapy, either alone (25.0%) or in combination with new irradiation (33.4%), was the most commonly introduced treatment.

**Figure 5:**
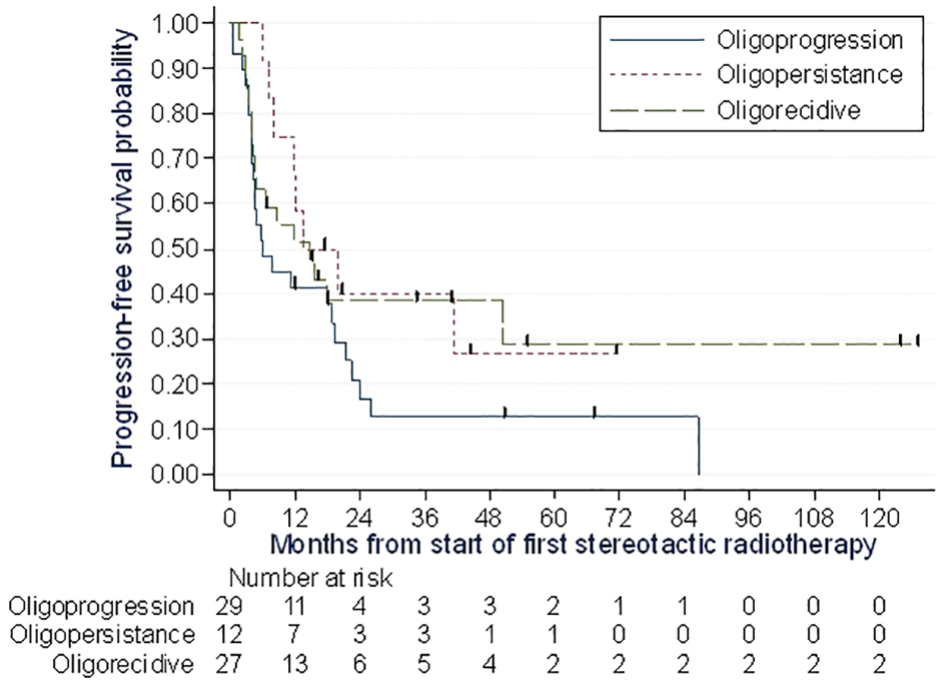
Progression free survival probability assessment of 69 melanoma patients at two cancer centers in Lille and Caen in France between 2007 and 2020, according to OMD status.

Concerning analyses carried out for the 3 different main oligometastatic indications: Among the 27 patients treated for oligorecurrence, with the aim of limiting or delaying the need for systemic therapies, the PFS at 1, 2 and 3 years was 51% (Min-Max: 31.0-68.0), 38% (Min-Max: 20.0-57.0) and 38% (Min-Max: 20.0-57.0) respectively (**Figure 5**). Twelve of these patients underwent SBRT combined with systemic treatment, with a 1-year PFS of 49% (Min-Max: 19.0-73.0). Fifteen patients underwent SBRT alone with a 1-year PFS of 53% (Min-Max: 26.0-74.0). After relapse, the median time to introduction of a new treatment was 3.8 months. They had SBRT alone because their disease was not aggressive enough, to avoid adverse effects, or because they had associated comorbidities limiting the systemic treatment.

Of the 29 patients treated for oligoprogression, with the aim of eliminating escape lesions that had lost sensitivity to systemic therapy, the PFS at 1, 2 and 3 years was 41% (Min-Max: 24.0-58.0), 17% (Min-Max: 5.0-33.0) and 13% (Min-Max: 3.0-28.0) respectively (**Figure 5**). Twenty patients continued with the same line of treatment. The median time to change treatment was 5 months (3.7-22.3) for the 9 patients who stopped immunotherapy for another line of treatment.

Among the 12 patients treated for oligopersistent lesions with the aim of discontinuing systemic therapy, the PFS at 1, 2 and 3 years was 58% (Min-Max: 27.0-80.0), 40% (Min-Max: 14.0-66.0) and 40% (Min-Max: 14.0-66.0) respectively (**Figure 5**). SBRT made it possible to stop systemic treatment in 7 patients, 4 of whom were on immunotherapy.

Most patients (82.6%) did not experience any adverse events, while 17.4% experienced acute, grade 1 (asthenia, nausea, cough and pain) or 2 (cough and hemoptysis) toxicity. Notably, no toxicities of grade 3 or higher were observed, either immediate or delayed. Furthermore, the combination of systemic therapies with SBRT did not lead to an increase in toxicity. No variables were significantly associated with OS in univariate analysis, nor with PFS or time to LC. All results of the study variables are included in **Supplementary Table 2**.

## Discussion

To our knowledge, this study is one of the first to solely evaluate the outcomes of SBRT for melanoma oligometastases. It is also one of the largest studies, given the number of patients, compared to previous studies. We provide evidence to supplement existing literature that shows SBRT is an effective and reliable treatment modality for OMDs and can be incorporated into the management of melanoma patients with extracranial oligometastases. The notion of oligometastases is a rapidly developing concept, and proof of the efficacy of SBRT in the therapeutic strategy for oligometastatic cancer has recently been demonstrated in randomized trials (12, 13). It is a focal treatment option on par with surgery or radiofrequency, which has become increasingly popular for treating oligometastatic cancers (20).

In regard to time to LC rates, our results concur with literature. Comparing the time to LC rate findings in a melanoma population to those of Franceschini et al. (2017) reveals that our study found a longer median follow-up time (42.6 months vs. 13 months) and a relatively similar time to LC rate at one year (94.2% vs. 96.6%). However, our findings reported a better time to LC rate at 2 and 3 years (90.3% vs. 83%) (16). Moreover, we described significantly higher time to LC rates compared to Stinauer et al. (2011), in which the time to LC rate was 82% at 1 year for 17 patients, and Kropp et al. (2016), in which the time to LC rates at 1 and 2 years were 83% and 63%, respectively for 16 patients treated with hypo-fractionated radiotherapy at sites of progression after treatment with ipilimumab (17, 18). In the prospective study enrolling 99 patients with different types of cancer, Palma et al. (2020) found the overall long-term time to LC rate was 63% for 99 patients treated with SBRT (13).

With reference to OS and PFS rates, we found higher numbers compared to Franceschini et al. (2017). They reported in an oligometastatic melanoma population, lower OS rates of 41% and 21% as well as lower PFS rates of 18.5% and 14% at 1 and 2 years, respectively. This could be partially attributed to a higher proportion of systemic therapies combined with SBRT in our study since 68% of patients received combination therapy in comparison to 29% in Franceschini et al. (16). Klemen et al. (2019) showed local therapy including radiotherapy for oligoprogression after immunotherapy can result in three-year PFS of 31% and five-year disease specific survival of 60% (19). These results are quite similar to ours.

A UK registry study of 1,442 patients treated with SBRT for extracranial oligometastases reported good OS rates, with variations depending on primary lesion site and a 2-year OS rate of 60.5% for melanoma patients (21). Randomized trials have also shown improved OS rates with the addition of SBRT to treatment for OMD including Non-Small Cell Lung Cancer (NSCLC) and prostate cancer (8–14). Livingstone et al. (2022) reported results in recurrence-free survival and overall survival for adjuvant nivolumab plus ipilimumab and adjuvant nivolumab alone in patients with stage IV melanoma with no evidence of disease after resection or radiotherapy. They found immunotherapy significantly improved 4-years RFS and OS compared with placebo (22). Finally, Gabani et al., highlighted that we must select the RT site (bone irradiation was associated with pore OS in this study) and the timing of RT (RT prior immunotherapy seems to improve OS) (23). In light of these studies and the rise of immunotherapy, the use of SBRT in the treatment strategy of oligometastatic melanoma, using as a closing treatment or in combination with immunotherapy, should be further explored.

The oligometastatic status of patients was reported according to the ESTRO-EORTC recommendations (7). The most common statuses were genuine *de novo* OMDs, metachronous, in oligorecurrence (in 24.6% of cases), and induced OMDs in oligoprogression in 21.7% of cases. Patients in the first category could potentially benefit from SBRT combined with systemic therapy from the onset allowing for a combined and aggressive therapeutic strategy, while those in the second category could benefit from SBRT in the second phase to control resistant clones and maybe restore sensitivity to systemic therapy allowing it to be continued in the same way.

This corresponds to two common clinical situations in practice, which can be found in other studies: In a retrospective study by Willmann et al. (2022) that included 385 oligometastatic patients treated with extracranial SBRT, the most common oligometastatic statuses were similar to those found in our study and also had comparable proportions (24). Patients with induced OMDs had a significantly shorter median OS (28.1 months) than those with *de novo* (46.3 months) or repeat (50.3 months) OMDs, although the median PFS was longer in *de novo* OMDs. *De novo* OMD was the most frequent status in Baker et al., 2022 where PFS rates differed significantly among the OMD groups (25). Patients with the synchronous OMD had the longest PFS rates, while patients with oligoprogression, especially induced oligoprogression, had the shortest rates and matched with Willmann et al. (24).

Looking at our oligometastatic subgroups: in oligoreccurence OMDs, SBRT doesn’t seem to allow to limit or delay the introduction of a systemic treatment. However, this result can largely be explained by the small size of the group. In oligoprogression OMDs, our results show that SBRT could maintain current lines of treatment by restoring sensitivity to systemic therapy. Finally, in case of oligopersistence OMDs, SBRT could be used as a closing treatment to stop systemic therapy. This subgroup seems to have a better PFS.

The median time to change systemic therapy among patients receiving a combination of systemic therapy and SBRT was 5.7 months. Since it could potentially restore sensitivity to the current systemic therapy by eliminating oligometastases resistant to the treatment line, this finding is particularly noteworthy in a disease with limited treatment options. Additionally, SBRT delayed the initiation of systemic therapy in 12 patients, thus sustaining quality of life. This strategy was investigated in the multicenter STOMP trial for prostate cancer as reported by Ost et al. (2018) (11).

We only found a small proportion (17.4%) of patients that experienced mild acute toxicity (grade 1 or 2), with none suffered severe toxicity (grade 3 or higher). This finding is consistent with previous research that indicates that SBRT is generally well tolerated. Nonetheless, we concluded that all patients with oligometastatic melanomas (regardless of the site treated, oligometastatic status or the presence of associated systemic treatment) may benefit from SBRT. Continued research in this area is therefore warranted and should consider including additional data such as the presence of liver metastases and LDH levels (particularly for metastatic melanoma patients undergoing immunotherapy). A recently published cohort study by Waninger et al. (2021) of 357 metastatic melanoma patients treated with immunotherapy showed that the presence of liver metastases in patients at the start of immunotherapy was significantly associated with patient survival. For instance, the median OS was 16.3 (3.5-28.8) months for patients with M1c stage cancer and liver metastases versus 56.5 (10.8-62.2) months for those without liver metastases. Similar results were found for the PFS (26). For LDH levels, Waninger et al. (2021) reported that patients with normal LDH levels had significantly better survival than those with high LDH levels (26).

In our study, of the 69 patients, 13 were alive and free of progression at 24 months after SBRT, while 51 reported progressions (44 being systemic). This may suggest the presence of subclinical metastases that were undetectable at the time of SBRT. To optimize the selection of patients, it may be necessary to continue with future studies on advanced imaging techniques and the identification of specific biomarkers for OMD (27–31). Currently, there have been no validated biomarkers available for clinical use, however, ongoing research on circulating tumor DNA and circulating tumor cells is promising. Kaddour et al., reported during SBRT increased following by undetectable levels of ctDNA (32). Future publications of randomized phase III trials (SABR COMET 3 and SABR COMET 10) are eagerly awaited to provide efficacy data related to SBRT in OMDs (33, 34). Additionally, the OligoCare study has included 1,500 patients to evaluate the classification of OMDs, and may potentially include melanoma patients to evaluate the benefit of adding SBRT to the treatment strategy for oligometastatic melanoma (35, 36).

In terms of strengths, our study population was larger than previous retrospective ones on SBRT used on melanoma oligometastases and was multicentric with a relatively long median follow-up (42.6 months). The homogeneity of our study compared to literature was also a key strength. Furthermore, the methodology used to report dosimetry data according to the recommendations of the ICRU 91 and the OMD classifications (ESTRO-EORTC criteria) was another strength in that it allowed us to harmonize data concerning patients treated for OMDs.

The main limitation of our study was its retrospective design, which may have introduced information and selection bias typical of such studies. The heterogeneity of our population can also be considered as a limitation given the inclusion of patients with different systemic treatments (immunotherapy, chemotherapy and targeted therapy), which is why we chose to perform a sensitivity analysis by estimating PFS excluding patients who had received targeted therapy or chemotherapy. Despite being conducted in two centers and having a larger study population, our cohort may be considered relatively small compared to other studies. This may have been because the use of SBRT in the treatment of melanoma metastases is not yet widely established in clinical practice. In addition, we chose to include only patients treated for secondary lesions in the lung and/or liver and/or lymph nodes, in order to homogenize our cohort as these are the main metastatic sites for melanoma. Brain oligometastases treated with SBRT were not included in our inclusion population due to the different treatment options for these. However, it is expected that studies focusing on a single histological type of cancer would also have a limited number of patients.

## Conclusion

In the treatment of oligometastatic melanoma metastases, we found that SBRT was an effective and reliable tool that enabled high time to LC rates on treated lesions and led to an encouraging OS. Alongside advancements in systemic therapies for melanoma, our study supports the continued use of SBRT for treating extracranial oligometastases in melanoma patients. SBRT could limit or delay the need for systemic therapies, maintain current lines of treatment in the case of oligoprogression, or serve as a closing treatment to stop systemic therapy. Future randomized studies are necessary in order to validate these findings and determine the role of SBRT in the therapeutic approach to oligometastatic melanoma.

## Data availability statement

The raw data supporting the conclusions of this article will be made available by the authors, without undue reservation.

## Ethics statement

Ethical approval was not required for the studies involving humans because Ethical approval for this study was not required. The study complies with the reference methodology MR004 adopted by the French Data Protection Authority (CNIL). None of the patients had objected to the use of their clinical data for research purposes. The studies were conducted in accordance with the local legislation and institutional requirements. Written informed consent for participation was not required from the participants or the participants’ legal guardians/next of kin in accordance with the national legislation and institutional requirements because Ethical approval for this study was not required. The study complies with the reference methodology MR004 adopted by the French Data Protection Authority (CNIL). None of the patients had objected to the use of their clinical data for research purposes.

## Author contributions

VT: Conceptualization, Investigation, Methodology, Project administration, Supervision, Writing – original draft, Writing – review & editing. SM: Conceptualization, Writing – review & editing. EB: Formal analysis, Methodology, Writing – review & editing. ML: Methodology, Writing – review & editing. EM: Investigation, Resources, Writing – review & editing. LV: Resources, Writing – review & editing. DP: Writing – review & editing. LM: Writing – review & editing. XM: Conceptualization, Investigation, Methodology, Supervision, Writing – review & editing.
