# Supplementary material for: Stereotactic body radiotherapy as a viable treatment on extracranial oligometastases in melanoma patients: a retrospective multicentric study
Source: Front Oncol. 2024 Mar 5;14:1322515. doi: 10.3389/fonc.2024.1322515 (PMC10949887; doi:10.3389/fonc.2024.1322515)
Supplement: Supplementary file 1 [file DataSheet_1.docx]

**Supplementary Material**


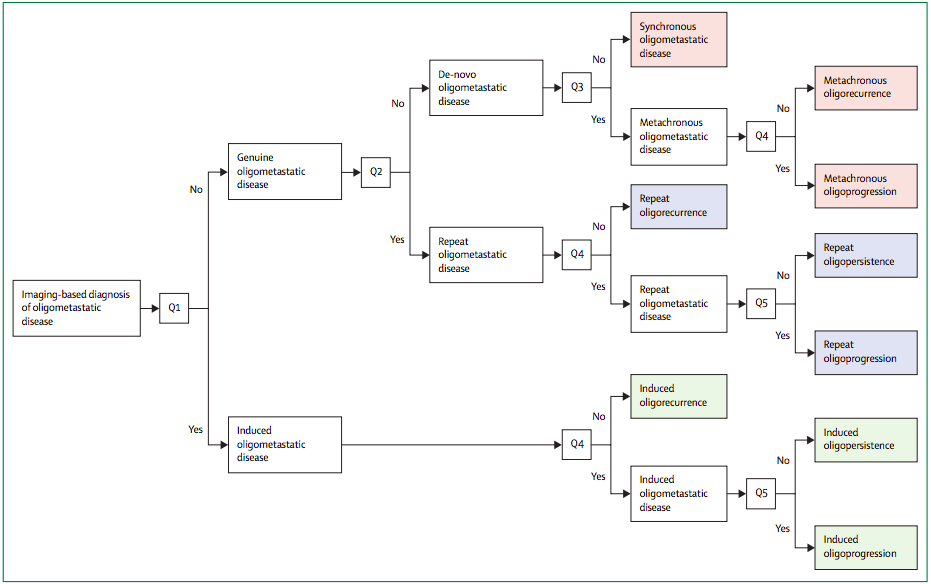


Q1; Does the patient have a history of poly-metastatic disease before the diagnosis of oligometastatic disease?

Q2; Does the patient have a history of oligometastatic disease before the diagnosis of oligometastatic disease?

Q3; Was oligometastatic disease first diagnosed more than six months after the diagnosis of the primary cancer?

Q4; Is the patient on active systemic therapy at the time of diagnosis of oligometastatic disease?

Q5; Are there any progressive oligometastatic lesions on current imaging?

**Supplementary Figure 1*.*** Decision tree for the classification of oligometastatic disease according to Guckenberger et al, (2020)

**Supplementary Table 1.** Dosimetric data

| **Volume** | **Total** | **Missing data** | **Mean**±**SD** | **Median (Min-Max)** |
| --- | --- | --- | --- | --- |
| **GTV D98** | 85 | 3 | 44.8±11.7 | 48.0 (15.2-73.7) |
| **GTV D50** | 85 | 3 | 48.5±12.3 | 51.8 (17.1-78.9) |
| **GTV D2** | 85 | 3 | 50.8±12.9 | 53.6 (17.8-81.9) |
| **CTV D98** | 18 | 70 | 44.8±7.7 | 46.6 (25.3-57.2) |
| **CTV D50** | 18 | 70 | 50.3±6.0 | 51.1 (31.0-63.0) |
| **CTV D2** | 18 | 70 | 53.7±6.2 | 54.5 (33.6-67.0) |
| **PTV D98** | - | - | 40.1±10.2 | 43.0 (13.1-66.5) |
| **PTV D50** | - | - | 46.2±11.3 | 49.2 (16.4-74.4) |
| **PTV D2** | - | - | 50.8±12.6 | 53.5 (17.8-81.6) |

GTV; gross tumor volume, CTV; Clinical target volume, PTV; Planned target volume

**Supplementary Figure 2.** Progression free survival probability assessment of 58 melanoma patients at two cancer centers in Lille and Caen in France between 2007 and 2020 (exclusion of the 11 patients with targeted therapy/chemotherapy)

**
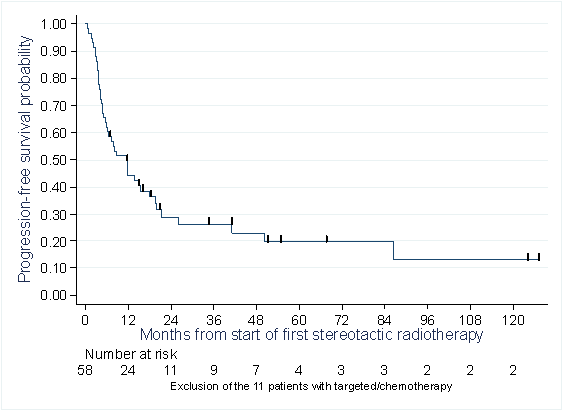
**

**Supplementary Table 2.** Association between efficacy criteria and patient and treatment characteristics

| **Association between factors**  **and effectiveness criteria** | **Overall survival** | | | **Progression free survival** | | | **Local control** | | |
| --- | --- | --- | --- | --- | --- | --- | --- | --- | --- |
|  | **Nb evnt/N** | **Univariate Analysis** | | **Nb evnt/N** | **Univariate Analysis** | | **Nb evnt/N** | **Univariate Analysis** | |
| **Overall population (n=69)** |  | **HR**  **[95% CI]** | **p-value** |  | **HR**  **[95% CI]** | **p-value** |  | **cs-HR**  **95% CI** | **p-value** |
| **SBRT sites** |  |  | 0.79 |  |  | 0.32 |  |  | 0.95 |
| *Hepatic* | 6/17 | 1 |  | 11/17 | 1 |  | 2/17 | 1 |  |
| *Pulmonary* | 11/28 | 1.01 [0.37 to 2.74] |  | 20/28 | 1.33 [0.63 to 2.80] |  | 2/28 | 0.74 [0.10 to 5.30] |  |
| *Lymph node* | 8/24 | 0.74 [0.26 to 2.17] |  | 20/24 | 1.75 [0.83 to 3.66] |  | 2/24 | 0.97 [0.14 to 6.93] |  |
| **OMD status** |  |  | 0.68 |  |  |  |  |  |  |
| *Genuine* | 16/47 | 1 |  | 34/47 | 1 | 0.29 | 3/47 | 1 | 0.22 |
| *Induced* | 9/22 | 1.08 [0.52 to 2.68] |  | 17/22 | 1.38 [0.77 to 2.48] |  | 3/22 | 2.72 [0.55 to 13.5] |  |
| **OMD status** |  |  | 0.26 |  |  | 0.49 |  |  | 0.45 |
| *Genuine, de novo* | 11/24 | 1 |  | 16/24 | 1 |  | 2/24 | 1 |  |
| *Genuine, repeat* | 5/23 | 0.41 [0.14 to 1.19] |  | 18/23 | 1.21 [0.62 to 2.37] |  | 1/23 | 0.55 [0.05 to 6.06] |  |
| *Induced* | 9/22 | 0.82 [0.34 to 1.98] |  | 17/22 | 1.52 [0.76 to 3.01] |  | 3/22 | 2.13 [0.35 to 12.8] |  |
| **OMD status** |  |  | 0.11 |  |  | 0.050 |  |  | * |
| *Synchronous* | 1/1 | 1 |  | 1/1 | 1 |  | 1/1 | 1 |  |
| *Metachronous* | 24/68 | 0.19 [0.02 to 1.44] |  | 50/68 | 0.13 [0.02 to 1.00] |  | 5/68 | * |  |
| **SBRT indication** |  |  | 0.45 |  |  | 0.08 |  |  | 0.81 |
| *Oligoprogression* | 10/29 | 1 |  | 25/29 | 1 |  | 3/29 | 1 |  |
| *Oligopersistence* | 4/12 | 0.87 [0.27 to 2.80] |  | 8/12 | 0.55 [0.25 to 1.22] |  | 1/12 | 0.50 [0.05 to 4.76] |  |
| *Oligorecurrence* | 10/27 | 1.03 [0.43 to 2.47] |  | 17/27 | 0.66 [0.35 to 1.22] |  | 1/27 | 0.35 [0.04 to 3.41] |  |
| *Synchronous OMD* | 1/1 | 5.31 [0.65 to 43.5] |  | 1/1 | 6.22 [0.76 to 50.6] |  | 1/1 | * |  |
| **SBRT indication - without considering synchronous OMD** |  |  | 0.97 |  |  | 0.22 |  |  | 0.62 |
| *Oligoprogression* | 10/29 | 1 |  | 25/29 | 1 |  | 3/29 | 1 |  |
| *Oligopersistence* | 4/12 | 0.88 [0.27 to 2.82] |  | 8/12 | 0.55 [0.25 to 1.22] |  | 1/12 | 0.50 [0.05 to 4.76] |  |
| *Oligorecurrence* | 10/27 | 1.03 [0.43 to 2.47] |  | 17/27 | 0.66 [0.35 to 1.22] |  | 1/27 | 0.35 [0.04 to 3.41] |  |
| **Total dose (Gy)** |  |  | 0.62 |  |  | 0.47 |  |  | 0.69 |
| *HR/1 Gy* | - | 1.01 [0.97 to 1.05] |  | - | 0.99 [0.97 to 1.02] |  | - | 0.99 [0.92 to 1.06] |  |
| **Dose per fraction (Gy)** |  |  | 0.59 |  |  | 0.31 |  |  | 0.56 |
| *HR/1 Gy* | - | 1.03 [0.92 to 1.15] |  | - | 0.96 [0.90 to 1.04] |  | - | 0.94 [0.76 to 1.16] |  |
| **Immunotherapy during SBRT** |  |  | 0.61 |  |  | 0.42 |  |  | 0.47 |
| *No* | 15/33 | 1 |  | 24/33 | 1 |  | 2/33 | 1 |  |
| *Yes* | 10/36 | 0.80 [0.35 to 1.84] |  | 27/36 | 1.26 [0.72 to 2.20] |  | 4/36 | 1.88 [0.34 to 10.3] |  |

OMD; oligometastatic disease, SBRT; stereotactic body radiotherapy, HR; hazards ratio, cs-HR; cause-specific hazard ratio, *Not estimated
